# Trait Anticipatory Pleasure Predicts Effort Expenditure for Reward

**DOI:** 10.1371/journal.pone.0131357

**Published:** 2015-06-26

**Authors:** Joachim T. Geaney, Michael T. Treadway, Luke D. Smillie

**Affiliations:** 1 Melbourne School of Psychological Sciences, The University of Melbourne, Melbourne, Australia; 2 Department of Psychology, Emory University, Atlanta, United States of America; Inserm, FRANCE

## Abstract

Research in motivation and emotion has been increasingly influenced by the perspective that processes underpinning the motivated approach of rewarding goals are distinct from those underpinning enjoyment during reward consummation. This distinction recently inspired the construction of the *Temporal Experience of Pleasure Scale* (TEPS), a self-report measure that distinguishes trait anticipatory pleasure (pre-reward feelings of desire) from consummatory pleasure (feelings of enjoyment and gratification upon reward attainment). In a university community sample (*N* = 97), we examined the TEPS subscales as predictors of (1) the willingness to expend effort for monetary rewards, and (2) affective responses to a pleasant mood induction procedure. Results showed that both anticipatory pleasure and a well-known trait measure of reward motivation predicted effort-expenditure for rewards when the probability of being rewarded was relatively low. Against expectations, consummatory pleasure was unrelated to induced pleasant affect. Taken together, our findings provide support for the validity of the TEPS anticipatory pleasure scale, but not the consummatory pleasure scale.

## Introduction

The desire to approach stimuli that give us pleasure has long been viewed as fundamental to human motivation and emotion [1], and impairment or dysfunction in the capacity to anticipate or experience pleasure—anhedonia—is a core feature of psychopathologies such as depression, schizophrenia, and Parkinson’s Disease [2–4]. Whilst it seems self-evident that people desire that which they enjoy, neurobiological studies of reward processing suggest that our motivation to approach rewarding stimuli or situations is dissociable from our experiences of pleasure and enjoyment upon reward consummation [5]. Recently, the *Temporal Experience of Pleasure Scale* (TEPS) was constructed in an attempt to distinguish between these two constructs in terms of stable trait characteristics [6]. Trait *anticipatory pleasure* refers to individual differences in the tendency to experience excitement, motivation, and desire in relation to future anticipated rewards. Conversely, trait *consummatory pleasure* refers to individual differences in the tendency to experience enjoyment, gratification, and contentment upon reward attainment. In this study we examine the extent to which these scales diverge in the prediction of reward-motivated behavior—specifically, the willingness to expend effort to obtain financial rewards—and the experience of experimentally induced pleasant affect.

### Anticipatory versus Consummatory Pleasure

The distinction between anticipatory and consummatory pleasure draws upon a number of distinct but related literatures. Chiefly, neurobiological studies on reward processing have demonstrated that the dopaminergic-based processes underlying the motivation or desire to seek or pursue reward, known as ‘wanting’, are dissociable from those underlying opioid-driven experience of pleasure or enjoyment during reward consummation, known as ‘liking’ [7–9]. Much of the primary evidence for this distinction comes from animal models. For instance, genetically engineered mice with elevated dopamine availability display hyperactivated approach behavior toward food [10], whereas dopamine-deficient rodents exhibit diminished approach behaviors, but both maintain normal affective taste reactivity to sweet substances [8,11]. On the other hand, reward enjoyment appears to be mediated in part by the opioid, endocannabinoid, and GABA systems within localized limbic and forebrain “hedonic hotspots” [9]. Consistent with the animal literature, human appetite research supports the dissociability of wanting and liking, especially in interaction with varying states of hunger and satiety [12,13]. Of clinical relevance, hyperactive wanting (without elevated liking) has been posited as a potential cause of obesity through overeating [14]. Similarly, addiction researchers have observed that substance-dependent individuals may crave and compulsively seek drugs known to have a diminished capacity to induce pleasure [15,16].

Another relevant literature concerns research into frontal cortical asymmetry using electroencephalography (EEG). This has supported a distinction between positive affective states that are higher versus lower in approach motivation intensity [17]. In particular, the work of Harmon-Jones and colleagues has demonstrated that greater left frontal cortical activity is associated with affective states characterized by approach motivation (e.g., anger or desire), independent of the extent to which such states are characterized by positively valenced feelings [18,19]. Among other implications, this work has prompted a reinterpretation of the negative relationship between left frontal asymmetry and depressive symptomatology. Specifically, it seems that this pattern of activity is related to the diminished experience of desire or motivation, rather than diminished feelings of pleasure (cf. [20]). More generally, this literature provides another example of the distinction between wanting/motivation processes and liking/affective processes.

Recently, clinical researchers have drawn upon this basic literature to propose a distinction between motivational and consummatory aspects of anhedonia [4,21]. Anhedonia is a key diagnostic criterion of Major Depressive Disorder in the *Diagnostic and Statistical Manual of Mental Disorders* (DSM-V), and has traditionally been defined in terms of diminished interest in and/or pleasure derived from things that were previously found rewarding [22]. This definition appears to conflate the motivation to engage in pleasurable pursuits with the experience of pleasure upon reward consummation [4]. The distinction between motivational and consummatory anhedonia was proposed to better recognize these dissociable processes, in line with the neurobiological literature reviewed above (e.g., [5,7,19]). In support of this distinction, a recent study found that individuals diagnosed with Major Depressive Disorder did not differ from healthy controls in consummatory liking of humorous cartoons (reward stimuli), but that consummatory liking only predicted motivation to re-view the cartoons amongst nondepressed persons [23].

### The Temporal Experience of Pleasure Scale (TEPS)

Drawing upon the aforementioned developments in basic and clinical research, Gard and colleagues (2006) developed the TEPS to provide a measure of stable individual differences in both anticipatory and consummatory pleasure experiences [6]. Anticipatory pleasure captures pleasure experienced in anticipation of a future reward or appetitive experience, as well as the capacity to imagine prospective rewards. In contrast, consummatory pleasure captures pleasant affective reactivity at the point of reward attainment or consummation. In line with the distinction between positive emotions that are high versus low in approach motivation intensity [17], anticipatory and consummatory pleasure are each positively valenced, but only anticipatory pleasure is held to relate to approach motivated affect (e.g., aroused or activated pleasant states). Finally, anticipatory and consummatory pleasure appear to closely (though inversely) map onto the notions of motivational and consummatory anhedonia.

In support of the validity of the TEPS, research to date has demonstrated associations of anticipatory pleasure with individual differences in reward motivation—measured by Carver & White’s (1994) Behavioral Activation System (BAS) scale [6,24–28]–hypomanic personality [25], and the gregariousness and excitement-seeking facets of extraverted personality [6]. Conversely, consummatory pleasure has been associated with (lower) physical anhedonia [6,28,29], reduced pleasantness ratings of pictorial stimuli [30], and the aesthetic appreciation facet of trait openness to experience [6]. In addition, a number of clinical studies have now shown that individuals diagnosed with schizophrenia report normal levels of consummatory pleasure but lower anticipatory pleasure—a finding that may reflect the motivational impairments observed in schizophrenia [28,30–33]. However, note that some studies have found the opposite pattern ([29,34], see also [35]), and others have found lower scores on both TEPS subscales [36,37].

Despite these generally encouraging findings, only very few validation studies have gone beyond self-report and/or cross-sectional methods to provide an experimental dissociation of anticipatory and consummatory pleasure (e.g., [38,39]). To further reduce this gap in the literature, the present study makes use of an effort-based behavioral paradigm for assessing reward motivation—the *Effort Expenditure for Rewards Task* (EEfRT) [40]. The EEfRT presents participants with a series of choices in which they may expend minimal effort to obtain a small financial reward or greater effort to obtain a larger reward under varying certainty of gain. The task was developed specifically to assess the motivational component of anhedonia, and modeled closely on rodent paradigms supporting the distinction between reward wanting and liking. Specifically, rats with impaired nucleus accumbens dopamine function have been shown to redirect instrumental behavior away from relatively effortful actions (e.g., barrier climbing or lever pressing) required to obtain food with high reward value, instead choosing less favored but freely available food [41–44]. In human research with an fMRI task that manipulates effort and reward values, activity in the dorsal anterior cingulate cortex and ventral striatum reflected effort-discounted reward valuations (reward divided by effort), suggesting these brain regions are involved in evaluations of reward benefits weighed against effort costs [45]. Similarly, the EEfRT examines choices between a low-effort task and a high-effort task as a function of trial-by-trial variations in reward magnitude and probability. In contrast to the rodent paradigms, however, trial-wise reward probability in the EEfRT applies categorically and equally to the low and high effort options. Therefore, the probability of reward attainment is not freely dependent on effort (providing trial completion). Consistent with findings from the animal paradigm, willingness to choose the high-effort task (for greater rewards) has been found to increase following *d*-amphetamine stimulation [46]. The proportion of hard-task choices is also correlated with amphetamine-induced striatal dopamine release assessed by Positron Emission Tomography [47], as well as left-frontal cortical asymmetry [48]–an established neural signature of approach motivation [19]. In both of these latter studies the relevant neural index predicted hard-task choices specifically on low probability trials. It is possible that the low probability condition is the most sensitive to individual differences in approach motivation due to greater associated probability costs. In line with these previous findings, we expect the anticipatory pleasure scale (a putative index of pre-reward anticipatory ‘wanting’) to predict greater willingness to engage in high-effort responding for reward, particularly for trials presenting a low probability of reward delivery. Conversely, we do not expect any relation between the consummatory pleasure scale (a putative index of reward ‘liking’) and responding during the EEfRT.

To assist in the validation of the consummatory pleasure scale, we employed a pleasant mood induction procedure used in recent research [49,50]. The stimuli in this procedure consist of short vignettes describing enjoyable situations (e.g., *you are walking through a quiet*, *picturesque forest*), in which participants imagine themselves as they listen to gentle orchestral music. Drug challenge studies have shown that affective responses to similar mood inductions are influenced by opioid receptor function [51,52]. Importantly, the imagery in these vignettes is low in motivational salience, and the procedure does not produce increases in approach-motivated positive affect [49]. Rather, this procedure has been shown to increase low-activation pleasant affect (e.g., feelings of contentment and satisfaction), which are representative of the feelings that accompany reward consummation. We therefore predict that consummatory pleasure will be associated with higher levels of pleasant affective reactivity during this mood induction procedure. Conversely, no such relationship should be observed for the anticipatory pleasure scale.

In sum, the current study aimed to build on the relatively few existing experimental validations of the TEPS questionnaire. To this end, we relate the two TEPS subscales to reward-directed behavior in the EEfRT task, and to the experience of pleasant affect in response to a mood induction procedure. We expect to observe a double dissociation, whereby anticipatory pleasure will predict increased effort-expenditure for reward, but should *not* be associated with the experience of induced pleasant affect, and consummatory pleasure should predict affective responses to the pleasant mood induction, but should *not* predict effort-expenditure for reward.

## Materials and Methods

### Participants

Ninety-seven participants (59% female) aged 18–44 (*M* = 23.87, *SD* = 6.37) were recruited via advertisements displayed around The University of Melbourne campus. All participants were informed they would be paid at least $5 for their time, which could be increased to A$15 (~ US$13.70) depending on task performance. In fact, all participants received the full A$15 to ensure equity. Participants completed measures reported previously by Smillie, Geaney, Wilt, Cooper, & Revelle (2013, study 2, [50]), however data concerning the EEfRT and the questionnaires used in this study (see below) have not been reported previously, and are locatable online (via doi.5061/dryad.nm13s).

### Procedure

Participants first provided written informed consent and demographic information. Next, the first of two counterbalanced sets of questionnaires was presented (including some not related to the aims of this study), followed immediately by the mood induction procedure, and then the second set of questionnaires. Finally, participants performed the EEfRT and a separate behavioral task not reported on here (counterbalanced).

### Ethics Statement

This research was approved by the Human Research Ethics Committee of The University of Melbourne, and conducted in accordance with the ethical guidelines set out by the National Health and Medical Research Council of Australia. All participants provided written informed consent prior to participation, and were debriefed at the end of the session.

### Questionnaires

#### Temporal Experience of Pleasure Scale (TEPS)

The TEPS [6] consists of 10 items to assess anticipatory pleasure (TEPS-ANT; e.g., *Looking forward to a pleasurable experience is in itself pleasurable*) and 8 items to assess consummatory pleasure (TEPS-CON; e.g., *The sound of crackling wood in the fireplace is very relaxing*). Responses were recorded on a Likert scale ranging from 1 (*very false for me*) to 6 (*very true for me*), from which mean scores were computed. Internal consistency as estimated by Cronbach’s alpha coefficients was satisfactory for the TEPS-ANT (α = .76) but somewhat low for the TEPS-CON (α = .54), which is consistent with some previous studies (e.g., internal consistency, α = .64 [6]; test-retest reliability, *r* = .48 [53]).

#### Additional trait measures

To strengthen our test of the convergent validity of the TEPS, we included the widely utilized BAS scale [24] and Snaith Hamilton Pleasure Scale (SHAPS; [54]). The BAS consists of 13 items concerning approach behavior and associated emotions (e.g., *I go out of my way to get things I want; When I see an opportunity for something I like*, *I get excited right away*) (α = .81). As such, it is conceptually related to anticipatory pleasure, and has correlated with the TEPS-ANT in prior research [6,27]. We therefore expect the BAS scale to converge with the TEPS-ANT in the prediction of effort-expenditure for reward. Conversely, the SHAPS comprises 14 items measuring the tendency to enjoy pleasant consummatory experiences (e.g., *I would enjoy seeing other people’s smiling faces*) (α = .83). The SHAPS and TEPS-CON are thus analogous such that they both focus on consummatory pleasure from attained rewards. We therefore expect the SHAPS to converge with the TEPS-CON in regards to affective response to the pleasant mood induction.

### Effort Expenditure for Rewards Task (EEfRT)

The EEfRT is a decision-making task that assesses willingness to expend physical effort for rewards, as contingent on varying reward magnitude and probability of attainment (see Treadway et al., 2009, for a detailed description [40]). In each trial, participants decide between an “easy-task” (chance to win $1.00) and a “hard-task” (chance to win between $1.24 and $4.30). The easy-task requires participants to use their first (index) finger of their dominant hand to make 30 successive keystrokes within 7 seconds (using the L or S key of a QWERTY keyboard for right vs. left handed participants, respectively). In contrast, the hard-task requires participants to use their fourth (“little”) finger of their nondominant hand to make 100 successive keystrokes within 21 seconds, and thus demands greater physical effort (using the S or L key of a QWERTY keyboard respectively for right vs. left handed participants). Critically, trial success does not guarantee that participants will win the money available in that trial. Rather, success is subject to low (12%), medium (50%), or high (88%) probability of reward delivery, randomly varying across trials. Note that, for each trial, the probability of reward delivery applies equally to both the low reward choice and the high reward choice (e.g., 50% chance of winning $1.00 versus 50% chance of winning $2.37). This enables the effect of reward magnitude to be examined separately from reward probability, and also minimizes the cognitive demands of the task. At the end of each trial, participants received feedback about whether or not they had won the money available in that trial. During verbal instruction, participants were informed that the EEfRT would run for 20 min and they would be paid 10% of their total winnings at the end of the experiment (*M* = $5.86, *SD* = 0.47). We modeled the likelihood of choosing the hard-task (vs. the easy-task) using generalized estimating equations (GEE) [55]. The GEE method can be applied to repeated binary data using a logistic link function and allows continuous variables to be included as predictors. All models included reward probability (categorical), reward magnitude (continuous), and trial number (continuous) as within-subjects variables.

### Pleasant mood induction

Mood induction stimuli consisted of short written vignettes paired with orchestral music, and were adapted from previous research [49,56]. Participants were presented with three vignettes describing pleasant and tranquil events free of approach-related content, and encouraged to elaborate on them with mental imagery. The vignettes were (1) *You are lying in the warmth of the sun on a tropical beach*, *with the sound of gentle waves in the background*; (2) *You unexpectedly run into a friend from school*. *You go for coffee and have a great conversation*; and (3) *You are walking peacefully through a quiet and picturesque forest*. The vignettes were each displayed for 2 min in an automated slideshow. Prior instructions encouraged participants to “think the thoughts and feel the feelings” that they would if the events were actually occurring (for full instructions see Smillie et al., 2012 [49]). Accompanying the vignettes via headphones was a recording of *Venus*, *the Bringer of Peace*, from *The Planets* (Op. 32), an orchestral suite composed by Gustav Holst (1874–1934) [57]. Such combined modality mood inductions have been shown to have a potent influence on affective states [58].

To assess affective responses to the pleasant mood induction we used items drawn from the Multidimensional States Questionnaire (MSQ) [59] and the 12-Point Affect Circumplex questionnaire (12-PAC) [60]. State pleasant affect was measured using four items that are frequently used to assess positively valenced states that are neither high nor low in arousal/activation. These were, *Happy*, *Content*, *Satisfied*, and *Pleased*, responses to which were internally consistent both at baseline (α = .87) and post mood induction (α = .88). State positive activation was assessed using four items that are representative of positively valenced states high in arousal/activation. These were, *Enthusiastic*, *Proud*, *Energetic*, and *Excited*, which also demonstrated acceptable internal consistency both at baseline (α = .76) and post mood induction (α = .81).

## Results

### Preliminary Statistics

#### Questionnaires

Descriptive statistics from the self-report data are detailed in Table 1. Consistent with past research, BAS scores were moderately correlated with the TEPS-ANT but not the TEPS-CON [6,25]. The SHAPS also correlated more strongly with the TEPS-ANT than the TEPS-CON, which was unexpected given the consummatory focus of SHAPS items. Relations among our affect scales were moderate in size, which is consistent with their putative locations in the affective circumplex. Specifically, pleasant affect and positive activation putatively differ by a 45-degree angle when represented geometrically [60], which corresponds closely to the observed correlation between our two post-induction affect measures of *r* = .46. Finally, the pattern of correlations between trait variables and state affect was mostly conceptually coherent. The TEPS-ANT was more consistently correlated with positive activation across time points, although equally correlated with pleasant affect and positive activation at baseline. Surprisingly, the TEPS-CON was not significantly correlated with either affect measure at either time point. Lastly, as expected, BAS correlated more strongly with baseline positive activation, whereas the SHAPS was more strongly associated with baseline pleasant affect.

**Table 1:** Descriptive Statistics and Inter-Correlations of Self-Report Variables.

	Variable	*M*	*SD*	1.	2.	3.	4.	5.	6.	7.
**1**	TEPS-ANT	4.35	0.73	−						
**2**	TEPS-CON	4.38	0.66	.34**	−					
**3**	BAS	41.89	4.85	.47**	.13	−				
**4**	SHAPS	3.56	0.32	.46**	.32**	.20*	−			
**5**	Pleasant Affect (pre)	3.03	0.60	.34**	.13	.22*	.38**	−		
**6**	Pleasant Affect (post)	3.16	0.61	.19	.04	.06	.23*	.50**	−	
**7**	Positive Activation (pre)	2.62	0.62	.35**	.07	.35**	.23*	.67**	.32**	−
**8**	Positive Activation (post)	2.25	0.69	.28**	−.001	.18	.07	.31**	.46**	.40**

*Note*. TEPS-ANT = TEPS Anticipatory Pleasure subscale; TEPS-CON = TEPS Consummatory Pleasure subscale; BAS = Behavioral Activation System scale; SHAPS = Snaith-Hamilton Pleasure Scale; Pre/post = before/after the mood induction.

* *p* < .05;

** *p* < .01

#### EEfRT—manipulation check

Valid EEfRT data were not obtained from three participants due to two instances of computer failure and one of noncompliance. All remaining participants (*n* = 94) chose a mixture of easy- and hard-tasks throughout the EEfRT (hard-task proportion *M* = .53, *SD* = .14), at a high completion rate (*M* = 99.4%). The total number of completed trials varied because participants performed the EEfRT for the same length of time (20 min) but differed in their profile of choices (range = 43 to 68, *M* = 54.79). For consistency, we analyzed only the first 43 trials completed by all participants. GEE model 1 examined main effects of reward magnitude, and medium and high reward probability in relation to low reward probability. We also modeled effects of the Reward Probability × Reward Magnitude interaction term that indexes mental computations of *expected value* (see Table 2, Model 1). Significant positive effects of reward probability, reward magnitude, and expected value were uncovered, indicating that participants were generally willing to expend greater effort for rewards that were larger in magnitude and more likely to be delivered. There was also a significant negative effect of trial number, which is routinely observed in studies using the EEfRT [47,48], potentially reflecting a fatigue effect. Trial number was therefore retained as a covariate in all subsequent GEE models.

**Table 2:** GEE Modeling of Predictors of Hard-Task Choice Likelihood in the EEfRT.

					95% CI		
		*χ* ^*2*^	*b*	*SE*	Lower	Upper	*p*
**Model 1**	Medium Probability^a^	8.64	0.69	0.23	0.23	1.15	.003
	High Probability^a^	11.01	1.31	0.40	0.54	2.09	.001
	Reward Magnitude	18.25	0.41	0.10	0.22	0.60	< .001
	Expected Value	23.84	0.88	0.18	0.52	1.23	< .001
	Trial Number	17.52	−0.02	0.00	−0.02	−0.01	< .001
**Model 2**	TEPS-ANT	1.54	0.14	0.11	−0.08	0.36	.215
	TEPS-CON	0.82	−0.13	0.15	−0.42	0.15	.365
**Model 3**	TEPS-ANT × Probability:	8.96					.030
	× Low	6.11	0.57	0.23	0.12	1.03	.013
	× Medium	0.03	−0.02	0.13	−0.27	0.23	.874
	× High	0.02	0.02	0.15	−0.27	0.31	.885
	TEPS-CON × Probability:	1.93					.587
	× Low	0.02	0.03	0.23	−0.42	0.48	.902
	× Medium	1.36	−0.20	0.17	−0.52	0.13	.243
	× High	1.04	−0.17	0.17	−0.51	0.16	.308
**Model 4**	TEPS-ANT × Magnitude	1.51	0.05	0.04	−0.03	0.12	.220
	TEPS-CON × Magnitude	0.62	−0.04	0.05	−0.14	0.06	.429
**Model 5**	TEPS-ANT × Exp. Value	1.71	0.08	0.06	−0.04	0.20	.192
	TEPS-CON × Exp. Value	0.00	0.00	0.07	−0.14	0.14	.990

*Note*. All models included reward probability (categorical), reward magnitude, and trial number as within-subjects variables. *χ* ^*2*^ = Wald chi-square; *b* regression coefficients are linear predictors of the likelihood of choosing the hard-task; CI = confidence interval; EEfRT = Effort Expenditure for Rewards Task; TEPS-ANT = TEPS Anticipatory Pleasure subscale; TEPS-CON = TEPS Consummatory Pleasure subscale; Exp. = Expected.

^a^Estimates were computed in relation to the low (12%) reward probability level, the parameters for which are therefore redundant.

#### Pleasant mood induction—manipulation check

To examine the effectiveness of the pleasant mood induction, we conducted a 2 (pre/post induction) × 2 (affect type) repeated-measures ANOVA. This revealed a significant two-way interaction, *F*(1, 96) = 48.07, *p* < .001, *η*_*p*_^*2*^ = 0.33, suggesting that the mood induction diverged in its influence on pleasant versus activated positive affect. Further analysis confirmed that pleasant affect increased from baseline, *F*(1, 96) = 4.21, *p* = .043, *η*_*p*_^*2*^ = .04, whereas positive activation decreased, *F*(1, 96) = 25.97, *p* < .001, *η*_*p*_^*2*^ = .21. This confirms the effectiveness of the pleasant affective stimuli for eliciting a pleasantly-valenced low activation affective state, synonymous with extant descriptions of reward consummation and gratification (see Table 1 for means).

### Main Analyses

#### Relationship between the TEPS and effort-expenditure for reward

To test our main hypotheses we performed a series of GEE models (see Tables 2 and 3) with self-report trait variables included as predictors (consistent with Treadway et al., 2009 [40]). Model 2 tested for main effects of the TEPS scales, revealing nonsignificant effects of both TEPS-ANT and TEPS-CON. Next, models 3–5 separately examined interactions between the TEPS scales and reward probability, reward magnitude, and expected value. In support of our predictions, the TEPS-ANT × Reward Probability interaction was significant. More specifically, TEPS-ANT positively predicted the likelihood of choosing the hard-task when the probability of reward delivery was low. In contrast, the TEPS-CON × Reward Probability interaction was nonsignificant, as were interactions between the TEPS scales and reward magnitude, and between the expected value interaction term. Thus, reward magnitude did not impact on the association between the TEPS scales and effort-based responding for reward.

**Table 3:** GEE Modeling of Predictors of Hard-Task Choice Likelihood in the EEfRT.

					95% CI		
		*χ* ^*2*^	*b*	*SE*	Lower	Upper	*p*
**Model 6**	BAS	2.80	0.03	0.02	−0.01	0.07	.094
	SHAPS	0.00	0.01	0.24	−0.46	0.47	.977
**Model 7**	BAS × Probability:	8.83					.032
	× Low	7.28	0.11	0.04	0.03	0.18	.007
	× Medium	1.17	0.02	0.02	−0.02	0.06	.279
	× High	0.43	−0.01	0.02	−0.06	0.03	.512
	SHAPS × Probability:	0.37					.946
	× Low	0.02	0.06	0.47	−0.85	0.98	.891
	× Medium	0.09	−0.07	0.24	−0.53	0.39	.770
	× High	0.04	0.05	0.26	−0.46	0.56	.849
**Model 8**	BAS × Magnitude	3.79	0.01	0.01	−0.00	0.03	.052
	SHAPS × Magnitude	0.02	−0.01	0.08	−0.17	0.14	.885
**Model 9**	BAS × Exp. Value	2.17	0.01	0.01	−0.01	0.03	.140
	SHAPS × Exp. Value	0.26	0.05	0.10	−0.15	0.25	.610
**Model 10**	Δ Pleasant Affect (PA)	0.17	0.06	0.14	−0.22	0.34	.679
**Model 11**	Δ PA × Probability:	1.31					.726
	× Low	0.88	0.24	0.25	−0.26	0.73	.349
	× Medium	0.05	0.03	0.15	−0.26	0.32	.826
	× High	0.14	−0.06	0.17	−0.39	0.26	.710
**Model 12**	Δ PA × Magnitude	0.16	0.02	0.05	−0.07	0.11	.686
**Model 13**	Δ PA × Exp. Value	0.02	0.01	0.07	−0.14	0.16	.900

*Note*. All models included reward probability (categorical), reward magnitude, and trial number as within-subjects variables. *χ* ^*2*^ = Wald chi-square; *b* regression coefficients are linear predictors of the likelihood of choosing the hard-task; CI = confidence interval; EEfRT = Effort Expenditure for Rewards Task; BAS = Behavioral Activation System scale; SHAPS = Snaith-Hamilton Pleasure Scale; Exp. = Expected; Δ PA = pleasant affect pre-to-post change score.

The next four models (6–9) paralleled those of the TEPS scales, but instead included the BAS and SHAPS scales as predictors. First, both the BAS scale and the SHAPS had nonsignificant main effects. Next, in line with predictions and mirroring the TEPS-ANT finding, a significant BAS × Reward Probability interaction was observed. Again, the BAS scale positively predicted the likelihood of choosing the hard-task in low reward probability trials. In contrast, the SHAPS × Reward Probability interaction was nonsignificant. Finally, the SHAPS × Reward Magnitude interaction was nonsignificant, whereas the BAS × Reward Magnitude interaction bordered on significance. This suggests that the positive influence of reward magnitude was somewhat dependent upon BAS scores but did not vary with SHAPS scores. Finally, in model 9, neither BAS nor SHAPS significantly interacted with expected value.

For descriptive purposes, simple nonparametric correlations between hard-task proportions and self-report trait variables are presented in Table 4. These echo the pattern of findings based on GEE modeling, showing that both TEPS-ANT and BAS were associated with making a greater proportion of hard-task choices on trials for which the probability of reward delivery was low.

**Table 4:** Zero-Order Correlations Between EEfRT Hard-Task Proportions and Trait Variables.

	Reward Probability		
Trait Variable	Low (12%)	Medium (50%)	High (88%)
TEPS-ANT	.251*	−.017	−.001
TEPS-CON	.178	−.144	−.060
BAS	.360**	.090	−.043
SHAPS	.115	.026	.030

*Note*. EEfRT = Effort Expenditure for Rewards Task; TEPS-ANT = TEPS Anticipatory Pleasure subscale; TEPS-CON = TEPS Consummatory Pleasure subscale; BAS = Behavioral Activation System scale; SHAPS = Snaith-Hamilton Pleasure Scale.

* *p* < .05;

** *p* < .01

#### Relationship between the TEPS and induced positive affect

We next employed hierarchical regression to examine the relations between trait variables and affective response to the pleasant mood induction. First, both TEPS scales were entered as predictors of induced pleasant affect after controlling for baseline pleasant affect and positive activation. The initial model after step-1 was significant, *R* ^*2*^ = 0.25, *F*(2, 94) = 15.82, *p* < .001. This reflected an influence of baseline pleasant affect on post-induction pleasant affect, *β* = .52, *t* = 4.30, *p* < .001, whereas baseline positive activation did not contribute to prediction, *β* = −.02, *t* < 1, *ns*. After step-2, the additional variance explained by the TEPS scales was nonsignificant, *R* ^*2*^_*ch*_ = .002, *F*_*ch*_ (2, 92) < 1, *ns*. Neither TEPS-ANT, *β* = .04, *t* < 1, *ns*, nor TEPS-CON, *β* = −.03, *t* < 1, *ns*, predicted induced pleasant affect. We then repeated this analysis, replacing the two TEPS scales with the BAS and SHAPS scales at step-2. Again, the additional variance explained was nonsignificant, *R* ^*2*^_*ch*_ = .004, *F*_*ch*_ (2, 92) < 1, *ns*, with neither the BAS scale, *β* = −.05, *t* < 1, *ns*, nor the SHAPS, *β* = .05, *t* < 1, *ns*, predicting induced pleasant affect. Note that results were substantively similar when each trait scale was entered separately as a predictor of induced pleasant affect.

We also examined relations between trait variables and post-induction positive activation. The initial model with baseline pleasant affect and positive activation entered as predictors was significant, *R* ^*2*^ = 0.16, *F*(2, 94) = 9.23, *p* < .001, reflecting the influence of baseline positive activation on post-induction positive activation, *β* = .35, *t* = 2.79, *p* = .006. Baseline pleasant affect did not contribute to prediction, *β* = .07, *t* < 1, *ns*. When the TEPS scales were added to this model at step-2, the increase to prediction was nonsignificant, *R* ^*2*^_*ch*_ = .03, *F*_*ch*_ (2, 92) = 1.62, *p* = .20. TEPS-CON was unrelated to post-induction positive activation, *β* = −.09, *t* < 1, *ns*. Interestingly, TEPS-ANT made a modest contribution to prediction that approached significance, *β* = .19, *t* = 1.77, *p* = .082. Repeating this analysis using the BAS and SHAPS scales at step-2, the additional variance explained was again nonsignificant, *R* ^*2*^_*ch*_ = .004, *F*_*ch*_ (2, 92) < 1, *ns*, with neither the BAS scale, *β* = .06, *t* < 1, *ns*, nor the SHAPS, *β* = −.05, *t* < 1, *ns*, predicting post-induction positive activation. Results were substantively similar when each trait scale was entered separately as a univariate predictor.

#### Relationship between effort-expenditure for reward and induced pleasant affect

To supplement our test of the divergent validity of the TEPS subscales, we also examined the relationship between reward-motivated decision-making in the EEfRT and affective response to the pleasant mood induction. We expected that increases in pleasant affect, as calculated by subtracting baseline pleasant affect from the post-induction scores (Δ pleasant affect), would be independent of hard-task EEfRT choices. In line with expectations, results from GEE models 10–13 (Table 3) indicated that the main effect of induced pleasant affect was nonsignificant, as were the interactions with reward probability and reward magnitude (all *p* values > 0.3).

## Discussion

A substantial literature in behavioral neuroscience has drawn a distinction between motivation to approach or pursue future rewards and emotional enjoyment of reward upon attainment or consumption. This suggests that our wanting of reward is separable from our liking of reward (5,7–9), and has potential implications for our understanding of mental disorders involving impaired reward processing, such as anhedonia [21]. The Temporal Experience of Pleasure Scale (TEPS) was designed to distinguish between long-term experiences of pre-reward motivational feelings of anticipatory pleasure and feelings of consummatory pleasure upon reward attainment [6]. In prior studies the TEPS scales have correlated in mostly coherent ways with other self-report measures of related constructs (e.g., [6,25,28]). Clinical research has also found impaired anticipatory pleasure in individuals with schizophrenia, although findings here have been mixed ([28,31,32], cf. [29,34]). To date, however, less attention has been given to the convergent and discriminant validity of the TEPS in nonclinical populations, using experimental manipulations of core processes surrounding reward motivation and emotion. To help address this gap in the literature, we examined the TEPS scales in relation to an effort-based task assessing reward motivation and a recently validated protocol for inducing pleasantly valenced affect. We expected a double dissociation, such that anticipatory pleasure would predict effort-expenditure for reward, whereas consummatory pleasure would predict pleasant affective reactivity.

In support of the validity of the TEPS-ANT, we found that scores on this measure predicted willingness to expend effort for reward in the EEfRT. Specifically, on trials for which the probability of reward delivery was low, high anticipatory pleasure was associated with a greater number of effortful hard-task choices made toward higher potential gains. This parallels previous studies examining the impact of reward motivation processes on EEfRT response behavior, such as the finding that administration of a dopamine agonist increased hard-task choices also when the probability of reward delivery was low [46]. Our finding suggests that anticipatory pleasure may be involved in processes that mitigate perceived effort costs when deciding whether to pursue relatively large but unlikely rewards [42–44]. This directly bears on goal-related decision-making in everyday life, whereby the pursuit of a particular goal will likely preclude another—especially when effort costs are high. Relevant to this idea are findings from related clinical studies using effort-based reward tasks, which have indicated abnormal processing of informative reward contingencies in schizophrenia [61–64], autism [65], depression [66,67], and bilateral basal ganglia damage [68]. In the present study, convergent validity was also shown by the fact that the Carver and White (1994) BAS scale [24]–a widely used self-report measure of reward motivation—strongly correlated with anticipatory pleasure and closely mirrored the relationship that anticipatory pleasure had with the EEfRT. In addition, a positive interaction between BAS and reward magnitude bordered on significance, suggesting that the influence of reward magnitude was stronger for those relatively high on BAS. In contrast, although consistent with prior research [47,48], it is difficult to interpret why no such interaction was observed of TEPS-ANT. Indeed, because effort spent on low probability rewards is by definition unlikely to be rewarded, one would expect discriminations between relatively low (e.g. $1.24) versus high (e.g., $4.30) rewards to be especially critical during low probability trials in the EEfRT. Exploratory analyses supported this interpretation. Specifically, BAS (*χ* ^*2*^ = 6.87, *b* = 0.03, *p* = .009) and TEPS-ANT (*χ* ^*2*^ = 5.64, *b* = 0.18, *p* = .018) each positively interacted with reward magnitude when the analyses were restricted to low probability trials, but not medium or high probability trials (all *p* values > 0.2). It is plausible that the range of reward values in the EEfRT is too narrow to elicit clear differential effects under higher probability levels. Finally, evidence for divergent validity was shown by the fact that neither TEPS-ANT nor BAS were related to increased experience of pleasant affect following a low-arousal mood induction procedure. Interestingly, although on average approach-related positive affect decreased after the non-appetitive mood induction procedure, there was a near-significant increase for individuals high in anticipatory pleasure. Taken together, these findings suggest that the TEPS-ANT reflects variation in reward motivation processes and, importantly, is not related to the propensity to experience pleasant affect.

Our findings were less supportive of the validity of the TEPS-CON. As expected, consummatory pleasure was unrelated to behavior during the EEfRT task, suggesting that it is unrelated to individual differences in reward motivation. Unexpectedly, however, this scale was also unrelated to the degree of pleasant affect experienced during a pleasant mood induction procedure. Identical observations were made for the well-established SHAPS measure of pleasure experiences. It is possible that individuals in our study were *already* in a highly pleasant affective state before the mood induction began, in which case, our failure to support our hypotheses relating to consummatory pleasure may be owing to a ceiling effect. If so, we might expect to observe a positive correlation between the TEPS-CON and pleasant affect at baseline, and an attenuated correlation after the pleasant mood induction. A pattern to this effect was observed for the SHAPS (although note that these two correlations did not differ significantly, *p* > .05), whereas the correlations between consummatory pleasure and pleasant affect were effectively zero at both time points. Therefore, a weak ceiling effect might potentially explain our null findings for the SHAPS but not for the TEPS-CON.

Overall, the current study provides partial support for the TEPS in the form of a single dissociation, rather than the predicted double dissociation. Our lack of support for the validity of the consummatory pleasure scale constitutes a null result that must be interpreted cautiously. Nevertheless, there are some additional observations that may raise concerns about this scale. Chiefly, estimates of reliability for the TEPS-CON were below commonly accepted margins, which has also been the case in some previous studies [6,53]. This suggests that the associations among individual scale items are weaker than would be expected if they are assessing a common construct [69]. This problem might be partly attributable to the heterogeneity of pleasure experiences described by the items of this scale, which cover multiple sensory modalities ranging from the sound of a crackling fire (item 2, aural), to the smell of cut grass (item 5, olfactory), and to the beauty of a fresh snowfall (item 13, visual). It is also noteworthy that other previous TEPS findings have consisted of a single dissociation akin to the present results [28,30]. This includes those from a laboratory study in which anticipatory pleasure predicted the expected enjoyment of chocolate, but consummatory pleasure was unrelated to subsequently experienced enjoyment of chocolate [38]. Additionally, two recently developed pleasure scales, the *Specific Loss of Interest and Pleasure Scale* (SLIPS) [70], and the *Anticipatory and Consummatory Interpersonal Pleasure Scale* (ACIPS) [71,72], each of which are conceptually related to both anticipatory and consummatory pleasure, are both considerably more weakly correlated with TEPS-CON relative to TEPS-ANT. This broader pattern of findings might be described in terms of weaker concurrent and predictive validity for the consummatory scale relative to the anticipatory scale of the TEPS. However, this tentative suggestion should be weighed against more promising validity data, such as the finding that TEPS-CON scores predict pleasantness ratings of pictorial stimuli [30].

Despite the encouragement our findings provide for the TEPS anticipatory pleasure scale, it should be acknowledged that the behavioral paradigm employed in this study for assessing effort-based responding for reward (the EEfRT) is relatively new. It would therefore be appropriate to provide a conceptual replication of the present findings using an alternative paradigm for assessing reward motivation (e.g., the Card Arranging Reward Responsivity Objective Test, or CARROT; [73]). Another valuable avenue for future research would be to examine the validity of the TEPS scales in the context of a single paradigm assessing anticipatory motivational processes and consummatory responses in relation to a single reward (cf. [38]). By experimentally controlling the object of reward, such a task could test the dissociability of wanting and liking without the potential confounds of reward differences or artifactual methodological differences. Sherdell, Waugh, & Gotlib (2012) recently assessed consummatory liking and motivational wanting of humorous cartoons in terms of self-reported enjoyment and effort-expenditure to re-view these stimuli [23]. This promising paradigm would benefit from further validation, including in relation to known neural indices of approach motivation, which is currently even less-well established than the EEfRT. In addition, other tasks have been developed for similar purposes within a neuroimaging context, such as the effort-based task used by Kurniawan and colleagues (2013) [74] and the Monetary Incentive Delay task [75], which researchers have used to separately examine neural responses to anticipated and received reward. Future research could similarly contrast neural responses during the pre-reward and reward-delivery phases of the EEfRT. Despite clear scope for improvement, the consistency of results from studies that have used the EEfRT is encouraging. Specifically, various measures and manipulations associated with reward motivation and dopamine function have now been linked with greater effort-expenditure for rewards—in particular, rewards that are relatively unlikely—as operationalized by the EEfRT. Individuals who have been administered a dopamine agonist [46], who appear to have a relatively more responsive dopamine system [47], who have higher resting left frontal cortical activity [48], or who score highly on self-report measures of reward motivation and anticipatory pleasure (current study), all appear willing to pursue rewards under conditions where the average individual is discouraged by low likelihood of reward attainment. The EEfRT may therefore be a valuable instrument to employ in future studies examining reward motivation and related constructs.

To conclude, the present study has provided mixed support for the validity of the Temporal Experience of Pleasure Scale or TEPS. As predicted, anticipatory pleasure and a conceptually similar measure of trait reward motivation predicted willingness to pursue relatively large but unlikely financial rewards. In line with the distinction between reward wanting and liking, consummatory pleasure and a conceptually similar measure of pleasure experience did not share these associations. Unexpectedly, these latter measures were also unrelated to induced feelings of pleasant affect—a null finding that should be interpreted cautiously, and thoroughly evaluated in extensions of the present study. Despite the lack of a clear double-dissociation between the TEPS subscales, the present findings suggest that the anticipatory pleasure scale provides a useful conceptualization of individual differences in reward motivation processes.
